# Clarithromycin Suppresses Human Respiratory Syncytial Virus Infection-Induced *Streptococcus pneumoniae* Adhesion and Cytokine Production in a Pulmonary Epithelial Cell Line

**DOI:** 10.1155/2012/528568

**Published:** 2012-06-12

**Authors:** Shin-ichi Yokota, Tamaki Okabayashi, Satoshi Hirakawa, Hiroyuki Tsutsumi, Tetsuo Himi, Nobuhiro Fujii

**Affiliations:** ^1^Departments of Microbiology, Sapporo Medical University School of Medicine, South-1, West-17, Chuo-ku, Sapporo 060-8556, Japan; ^2^Department of Pediatrics, Sapporo Medical University School of Medicine, South-1, West-16, Chuo-ku, Sapporo 060-8543, Japan; ^3^Department of Otolaryngology, Sapporo Medical University School of Medicine, South-1, West-16, Chuo-ku, Sapporo 060-8543, Japan

## Abstract

Human respiratory syncytial virus (RSV) sometimes causes acute and severe lower respiratory tract illness in infants and young children. RSV strongly upregulates proinflammatory cytokines and the platelet-activating factor (PAF) receptor, which is a receptor for *Streptococcus pneumoniae*, in the pulmonary epithelial cell line A549. Clarithromycin (CAM), which is an antimicrobial agent and is also known as an immunomodulator, significantly suppressed RSV-induced production of interleukin-6, interleukin-8, and regulated on activation, normal T-cell expressed and secreted (RANTES). CAM also suppressed RSV-induced PAF receptor expression and adhesion of fluorescein-labeled *S. pneumoniae* cells to A549 cells. The RSV-induced *S. pneumoniae* adhesion was thought to be mediated by the host cell's PAF receptor. CAM, which exhibits antimicrobial and immunomodulatory activities, was found in this study to suppress the RSV-induced adhesion of respiratory disease-causing bacteria, *S. pneumoniae*, to host cells. Thus, CAM might suppress immunological disorders and prevent secondary bacterial infections during RSV infection.

## 1. Introduction

Human respiratory syncytial virus (RSV) is one of the most important infectious agents causing acute lower respiratory tract illness, such as bronchiolitis and pneumonia, in infants and young children [1, 2]. Viral RNA generated during RSV replication is recognized by host pattern recognition molecules, such as Toll-like receptor 3 (TLR3) and retinoic acid inducible gene-I (RIG-I), and it induces type I and type III interferon [3, 4]. Transcriptional induction of proinflammatory cytokines, chemokines, and interferons is mediated by NF-*κ*B and interferon regulatory factors (IRFs) [5, 6]. These mediators are believed to contribute to the pathophysiology of RSV infection, such as mucous hypersecretion, swelling of submucous, and infiltration of lymphocytes, neutrophils, eosinophils, and macrophages [7].

Frequently, there are coinfections with respiratory viruses, including RSV, and bacteria that cause community-acquired respiratory diseases, such as *Streptococcus pneumoniae* and *Haemophilus influenzae*. There is evidence for a positive correlation between infections with *S. pneumoniae *and RSV in the pathogenesis of otitis media, pneumonia, and meningitis [8–11]. *S. pneumoniae* and *H. influenzae* colonize to the host respiratory epithelium via host cell surface receptors, such as the platelet-activating factor (PAF) receptor [12–14]. These bacteria interact with the PAF receptor via phosphorylcholine, which is a component of the bacterial cell surface. Both live and heat-killed* S. pneumoniae* cells show an increased adhesion to human epithelial cells infected with RSV [15]. The upregulation of PAF receptor expression that is induced by respiratory virus infections, including those caused by RSV, results in the enhanced adhesion of *S. pneumoniae* and *H. influenzae* to respiratory epithelial cells [15–17]. PAF receptor expression and *S. pneumoniae* cell adhesion are also upregulated by exposure to acid, which causes tissue injury and an inflammatory response [18].

Clarithromycin (CAM) is 14-membered ring macrolide antibiotic that also acts as a biological reaction modifier with anti-inflammatory properties. In Japan, CAM is applied to diffuse panbronchiolitis, chronic bronchiolitis, otitis media, and chronic sinusitis as an immunomodulator [19–21]. The anti-inflammatory mechanism of CAM has not yet been completely clarified, but one of the important mechanisms for its anti-inflammatory action is considered to be the suppression of NF-*κ*B [22–24]. 

Recently, we reported that fosfomycin, which is an antibiotic, suppressed RSV-induced interleukin (IL)-8, regulated on activation, normal T-cell expressed and secreted (RANTES), and the PAF receptor by suppressing NF-*κ*B activity [25, 26]. On the other hand, Wang et al. report that CAM suppressed rhinovirus-induced *Staphylococcus aureus* and *H. influenzae *adhesions to nasal epithelial cells [27]. So we anticipate that CAM suppresses RSV-induced bacterial adhesion to epithelial cells, because expression of PAF receptor is controlled by NF-*κ*B [28, 29]

In the present study, we examined the effect of CAM on cytokine production, PAF receptor expression, and RSV infection-induced *S. pneumoniae *adhesion to respiratory epithelial cells.

## 2. Materials and Methods

### 2.1. Viruses, Cell Lines, Bacteria, and Reagents

RSV strain Long, human type II pulmonary epithelial cell line A549 and *S. pneumoniae* strain R6 were obtained from the American Type Culture Collection (ATCC, Manassas, VA). RSV was grown in HEp-2 cells. The virus titer of RSV was determined using a plaque-forming assay with HEp-2 cells as the indicator cells [25]. RSV infection to A549 cells was performed at multiplicity of infection (MOI) of 1. CAM was donated by Abbott Japan (Tokyo, Japan). A PAF receptor antagonist, 1-*O*-hexadecyl-2-acetyl-*sn*-glycero-3-phospho(N,N,N,-trimethyl)-hexanolamine, was purchased from Calbiochem-Merck (Darmstadt, Germany). An NF-*κ*B inhibitor, pyrrolidine dithiocarbamate (PDTC), was purchased from Sigma-Aldrich (St. Louis, MO).

### 2.2. Measurement of Cytokine Production

A549 cells were infected with RSV at MOI of 1. After 24-hour infection, culture supernatants of RSV-infected and -uninfected cells were collected. The amounts of IL-6, IL-8, and RANTES in the culture supernatants were determined by enzyme-linked immunosorbent assay (ELISA) (DuoSet ELISA development kit, R&D systems, Minneapolis, MN).

### 2.3. Reverse Transcription-Polymerase Chain Reaction (RT-PCR)

Semiquantitative RT-PCR was carried out as described previously [4, 30].

### 2.4. Flow Cytometry

 The cell surface expression of the PAF receptor was examined by flow cytometry as previously described [26]. The cells were harvested from culture flasks using a cell scraper and then incubated with 2.5 *μ*g/mL of mouse anti-PAF receptor monoclonal antibody (11A4 (clone 21); Cayman Chemical, Ann Arbor, MI) or mouse IgG2a,  *κ* isotype control antibody (eBioscience, San Diego, CA). After incubation at 4°C for 30 min, cells were collected by centrifugation and washed once with Dulbecco's phosphate-buffered saline (PBS (−)). Cell suspensions were incubated with a phycoerythrin-conjugated goat anti-mouse IgG F(ab)_2_ fragment antibody (1 : 100 dilution) (Abcam, Cambridge, UK) at 4°C for 30 min, and the stained cells were assessed with FACSCalibur (BD Bioscience, San Jose, CA).

### 2.5. Bacterial Adhesion Assay

*S. pneumoniae* adhesion was assayed using fluorescein-isothiocyanate- (FITC-) labeled *S. pneumoniae* as previously described [26]. Briefly, a bacterial suspension in 0.1 M NaCl-50 mM sodium carbonate buffer (pH9.5) at 1 × 10^8^ CFU/mL was prepared. FITC isomer-I (Dojindo Laboratories, Kumamoto, Japan) was added at a concentration of 1 mg/mL, and the mixture was incubated at 4°C for 1 h. The cells were washed three times with PBS (−).

CAM was added to monolayers of A549 cells 1 h prior to RSV infection. The A549 cells infected with RSV at an MOI of 1 for 24 h and uninfected A549 cells were incubated with FITC-labeled *S. pneumoniae *cells at MOI of 10 for 30 min at 37°C. For the control experiments, either 20 *μ*g/mL of the PAF receptor antagonist or 10 *μ*g/mL of the mouse anti-PAF receptor monoclonal antibody (11A4(clone 21)) was added to the A549 cells 1 h prior to the addition of the FITC-labeled bacteria. The cell monolayer was gently washed three times with PBS (−) and observed by fluorescence microscopy. Alternatively, the cells were harvested with cell scraper and then assessed by flow cytometry as previously described [26].

## 3. Results

**Figure 1:**
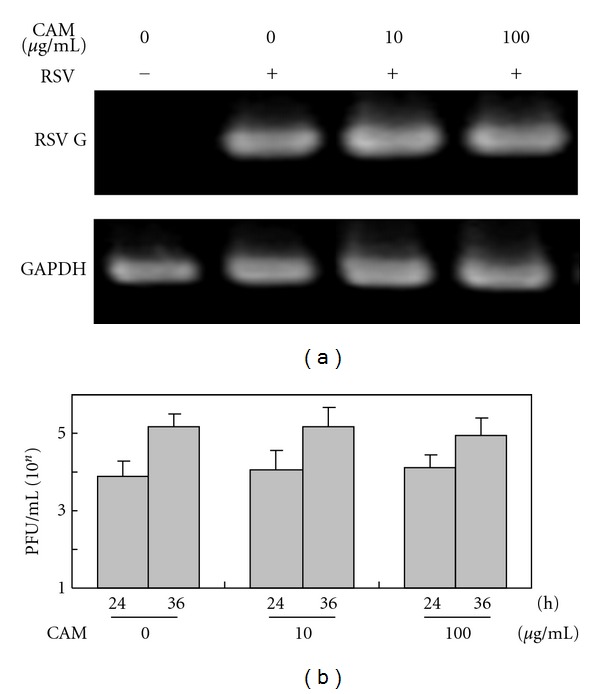
Effects of CAM on RSV G mRNA expression (a) and production of infectious virus particles (b) in A549 cells infected with RSV. One hour before RSV infection, CAM was added to A549 cell culture at the indicated concentration. A549 cells were infected with the RSV at MOI of 1. (a) RT-PCR. After 24 h of infection, total RNAs were extracted from the cells. The mRNA levels of RSV G were determined by RT-PCR. The mRNA levels of human glyceraldehyde-3-phosphate dehydrogenase (GAPDH) were carried out as a control. (b) Plaque-forming assay. After 24 h and 36 h infection, the culture supernatants were corrected. Virus titers in the supernatants were determined by plaque-forming assay using Hep-2 cells as the indicator cell. Each experiment was performed in quadruplicate. The mean value and standard deviation are shown.

 First, we examined the effect of CAM on RSV replication in A549 cells. RSV infection to A549 cells was performed at MOI of 1. After 24 and 36 h of infection, significant alterations of the RSV titers or expression levels of G mRNA were not observed by the addition of CAM even at a concentration of 100 *μ*g/mL (Figure 1).

**Figure 2:**
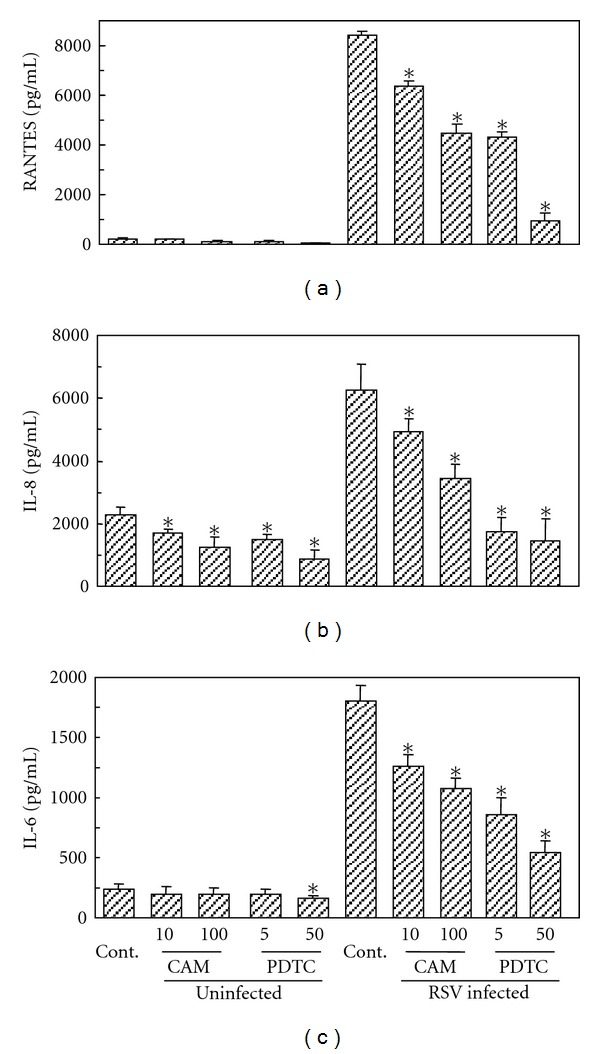
Effects of CAM and PDTC on RSV-induced RANTES (a), IL-8 (b), and IL-6 (c) production in A549 cells. One hour before RSV infection, CAM or PDTC is added to A549 cell culture at the indicated concentration. A549 cells were infected with the RSV at MOI of 1. After 24 h of infection, the culture supernatants were collected, and each cytokine in the supernatants was determined by ELISA. The experiments were performed in triplicate. The mean value and standard deviation were calculated. Statistical difference was examined by Student's *t*-test. *P < 0.01 compared to cytokine production without any reagent treatment in uninfected cells and RSV-infected cells, respectively.

When A549 cells were infected with RSV at MOI of 1, RANTES, IL-8, and IL-6 were markedly induced. These cytokine inductions were significantly suppressed in the presence of CAM in a dose-dependent manner (Figure 2). The degree of suppression by CAM was less than that by an NF-*κ*B inhibitor, PDTC.

**Figure 3:**
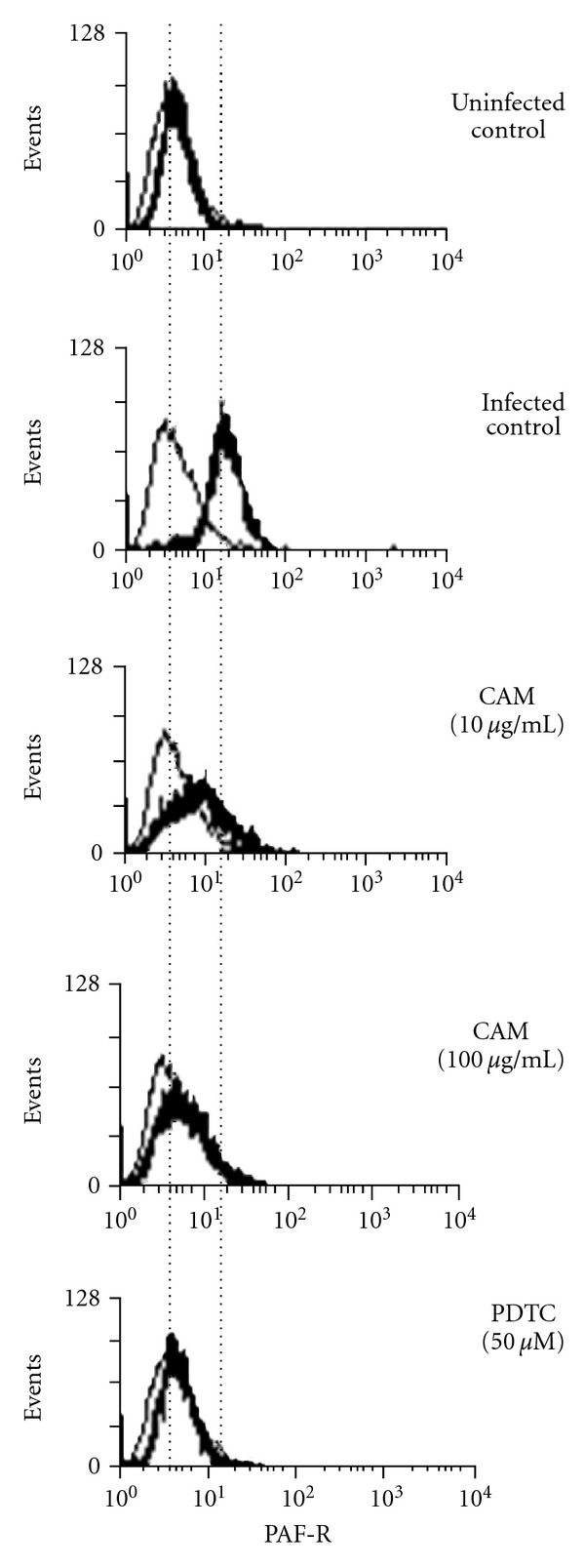
Effect of CAM and PDTC on RSV-induced PAF receptor expression in A549 cells. One hour before RSV infection, CAM or PDTC is added to A549 cell culture at the indicated concentration. The cells were infected with the RSV at MOI of 1. After 24 h of infection, the cells were collected and then stained with an anti-PAF receptor antibody and phycoerythrin-labeled anti-mouse IgG antibody (thick lines). The stained cells were analyzed by flow cytometry. Thin lines indicate the cells stained with an unrelated isotype control antibody instead of the anti-PAF receptor antibody.

PAF receptor expression on the cell surface is upregulated during RSV infection in A549 cells [26]. The RSV-induced upregulation of the PAF receptor was significantly suppressed by CAM and PDTC in a dose-dependent manner (Figure 3). The degree of suppression by CAM was slightly less than that by PDTC. Suppression of the PAF receptor expression was also observed when A549 cells were posttreated with CAM (4 or 12 h after RSV infection) (data not shown).

**Figure 4:**
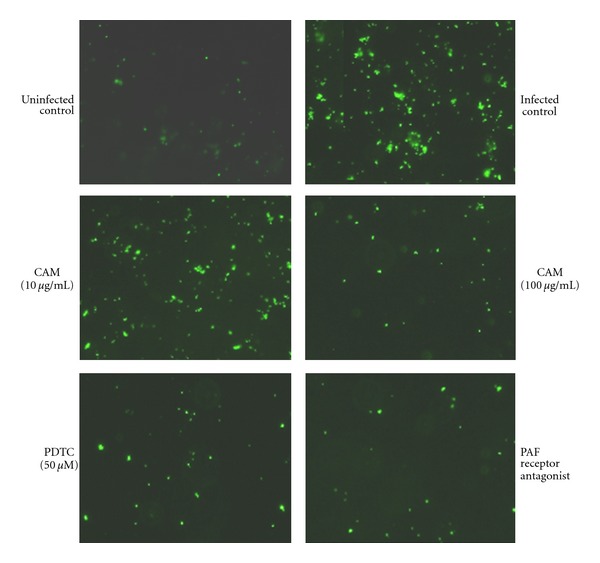
Suppression by CAM of RSV-induced adhesion of FITC-labeled *S. pneumoniae* to A549 cells, as observed by fluorescence microscopy. One hour before RSV infection, CAM (10 or 100 *μ*g/mL) or PDTC (50 *μ*M) was added to A549 cell monolayer. The cells were infected with RSV at MOI of 1. After 24 h of infection, FITC-labeled bacterial cells were added to the cell monolayer at MOI of 10, and incubation was continued at 37°C for 30 min. A PAF receptor antagonist (20 *μ*g/mL) was added to the cell monolayer 1 h before the addition of labeled bacterial cells. The bacteria adhering to the A549 cell monolayer were visualized by fluorescence microscopy.

**Figure 5:**
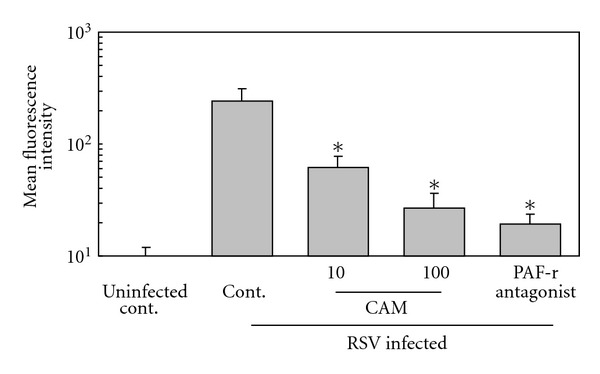
Suppression by CAM of RSV-induced adhesion of FITC-labeled *S. pneumoniae* to A549 cells, as observed by flow cytometry. Experiments were performed as in Figure 5. The A549 cell monolayer incubated with FITC-labeled *S. pneumoniae *cells was harvested by cell scraper and then applied to flow cytometry. Each experiment was performed in triplicate. The data present as mean value ± standard deviation of the mean relative fluorescence intensity. *P < 0.01 compared to RSV-infected cells without any reagent treatment.

We examined the adhesion of FITC-labeled *S. pneumoniae* cells to A549 cells by fluorescence microscopy (Figure 4) and flow cytometry (Figure 5). RSV infection significantly enhanced the adhesion of *S. pneumoniae* to A549 cells, and this enhancement was suppressed by adding a PAF receptor antagonist (Figures 4 and 5) or anti-PAF receptor monoclonal antibody (data not shown). This result indicated that the RSV-induced *S. pneumoniae *adhesion occurs via the PAF receptor on A549 cells. The bacterial adhesion was significantly suppressed by CAM, as well as PDTC. 

These lines of evidence confirmed that the expression of the PAF receptor was induced by RSV infection and indicated that this induction, and subsequent RSV-induced *S. pneumoniae* adhesion, can be suppressed by CAM treatment. 

## 4. Discussion

Macrolides, with the exception of the 16-membered ring type, have both anti-inflammatory and antibacterial functions [20, 21]. One of the important mechanisms of anti-inflammatory action is the suppression of NF-*κ*B activation [22–24]. Our recent studies show that RSV upregulates proinflammatory cytokines, such as IL-6, and chemokines, such as IL-8 and RANTES, in the respiratory epithelial cell line A549. Furthermore, the induction of chemokines by RSV is significantly suppressed by an antibiotic, fosfomycin, via suppression of NF-*κ*B activation [25]. In the present study, CAM was shown to suppress IL-6, IL-8, and RANTES, which are induced by RSV infection, at concentrations of 10 and 100 *μ*g/mL. Patel et al. reported that the concentration of CAM in fluid of the bronchopulmonary epithelial lining was 34.2 ± 5.16 *μ*g/mL at 4 h, 23.01 ± 11.9 *μ*g/mL at 12 h in healthy adults orally administered CAM 500 mg [31]. We observed that CAM did not affect RSV replication even at a concentration of 100 *μ*g/mL. However, it is reported that respiratory virus, such as RSV [32], rhinovirus [33, 34], and influenza virus [35], replication is suppressed by 14-membered ring macrolides, including CAM. The reasons of contradictory results between the report of Asada et al. [32] and our present study have been unclear. These two studies used different types of epithelial cells and different experimental conditions of RSV infection. Asada et al. used primary human tracheal epithelial cells, and in contrast we used A549 cell line. Asada et al. carry out infection at a lower titer of RSV (10^−3^ TCID_50_/cell) and measuring virus titer at a longer period (3–5 days) after infection. Our results indicated that suppression of the RSV-induced cytokines by CAM was not caused by the amount of replicated RSV. In other words, CAM was suggested to have suppressive activity of cytokine production independent of viral replication. Both IL-8 and RANTES, which are strongly upregulated during RSV infection, play important roles in pathogenesis [36, 37]. IL-8 primarily activates neutrophils and promotes their migration. RANTES is secreted from respiratory epithelial cells and promotes migration of eosinophils, basophils, monocytes, and neutrophils. In particular, RANTES is an efficient eosinophil chemoattractant involved in the pathogenesis of asthma [38]. CAM has been suggested to suppress the inflammatory disorders induced by RSV.

 In the present study, we also observed that CAM suppressed enhanced *S. pneumoniae* adhesion by RSV infection in A549 cells. The RSV-induced *S. pneumoniae* adhesion was mainly mediated by host PAF receptor, as indicated by that suppressed by the PAF receptor antagonist and anti-PAF receptor monoclonal antibody. The PAF receptor acts as a receptor for *S. pneumoniae* and *H. influenzae* [12–14]. Transcription of the PAF receptor gene is controlled by NF-*κ*B [28, 29]. We confirmed it by that the RSV-induced PAF receptor expression and *S. pneumoniae *adhesion were suppressed by an NF-*κ*B inhibitor, PDTC. We revealed that CAM also suppressed PAF receptor expression induced by RSV infection and *S. pneumoniae* adhesion to RSV-infected A549 cells. It should be caused by the suppression of NF-*κ*B activated by RSV infection. Recently, Wang et al. [27] reported that CAM suppressed rhinovirus-induced *S. aureus* and *H. influenzae* adhesions to nasal epithelial cells. They show that the expressions of fibronectin and carcinoembryonic antigen-related cell adhesion molecule (CEACAM), which act as receptors for *S. aureus* and *H. influenza,* respectively, are induced by rhinovirus and suppressed by CAM. The present study indicated that CAM suppressed the PAF receptor-phosphorylcholine (host-bacteria) interaction, which is enhanced by RSV infection, by inhibiting PAF receptor expression. CAM showed more potent suppression of RSV-induced *S. pneumoniae* adhesion and production of proinflammatory cytokines and chemokines than fosfomycin, as we reported previously [25, 26]. Notably, CAM significantly suppressed RSV-induced IL-6 production, whereas fosfomycin did not significantly [25]. This finding may be caused by that CAM is more potent than fosfomycin; however, the actual reason for this disparity is not clear. The upregulation of PAF receptor expression and the enhanced adhesion of pathogenic bacteria, such as *S. pneumoniae*, to respiratory epithelial cells are considered to be a major risk factor for secondary bacterial infections after primary respiratory viral infections. CAM may suppress both secondary bacterial infections and immunological disorders induced by RSV, without suppressing viral replication. Infection with other respiratory viruses, such as human parainfluenza virus 3 [16] and rhinovirus [17], also upregulates known receptors for the pathogenic bacteria, including PAF receptor and *S. pneumoniae* adhesion. On the other hand, influenza virus does not upregulate the known receptors for bacteria, whereas bacterial adhesion is increased by the infection [16]. McCullers [39] reported that influenza-induced bacterial adhesion to A549 cells was not inhibited by PAF receptor antagonist, and the PAF receptor knock-out mice did not show lower susceptibility to experimental secondary pneumonia caused by *S. pneunimoae* following influenza infection compared to the parent mice. Lines of evidence suggest that adherent inducing mechanisms of *S. pneumoniae* to host respiratory epithelial cells are varied among viruses. So CAM may not always suppress virus-induced pathogenic bacteria adhesion.

## 5. Conclusions

We proposed that clarithromycin efficiently suppressed PAF receptor-mediated *Streptococcus pneumoniae* adhesion to respiratory epithelial cells as well as RSV-induced proinflammatory cytokine and chemokine production. Clarithromycin may suppress secondary bacterial infections and immunological disorders during RSV infection.

## Abbreviations

CAM: Clarithromycin
ELISA: Enzyme-linked immunosorbent assay
FITC: Fluorescein isothiocyanate
IL: Interleukin
MOI: Multiplicity of infection
PAF: Platelet-activating factor
PDTC: Pyrrolidine dithiocarbamate
RANTES: Regulated on activation, normal T-cell expressed and secreted
RSV: Human respiratory syncytial virus
RT-PCR: Reverse transcription-polymerase chain reaction.
